# Protective activity of kudzu (*Pueraria thunbergiana*) vine on chemically-induced hepatotoxicity: in vitro and in vivo studies

**DOI:** 10.1186/s12906-016-1023-2

**Published:** 2016-01-29

**Authors:** Bo Yoon Chang, Dong-Sung Lee, Jun-Kyoung Lee, Youn-Chul Kim, Hyoung-Kwon Cho, Sung Yeon Kim

**Affiliations:** 1 Institute of Pharmaceutical Research and Development, College of Pharmacy, Wonkwang University, Shinyoung-dong 344-2, Iksan, Jeonbuk 570-749 Republic of Korea; 2 Department of Biomedical Chemistry, College of Health and Biomedical Science, Konkuk University, Chung-Ju, 27478 Republic of Korea; 3 Han Poong Pharmaceutical Co., Ltd, 333-24 1st Palbok-dong, Deokjin-gu, Jeonju-si, Jeonbuk 561-841 Republic of Korea

**Keywords:** Kudzu vine, Anti-oxidant, Hepatoprotection, *t*-BHP, CCl_4_

## Abstract

**Background:**

Kudzu (*Pueraria thunbergiana*) root has long been used in Traditional Chinese Medicine. However, the vine of the kudzu plant has been considered waste material. This study aimed to investigate the hepatoprotective properties of the kudzu vine.

**Methods:**

We created 0 %, 30 %, 70 %, and 95 % ethanolic kudzu vine extracts. The isoflavone contents of kudzu vine extract were quantified by high-performance liquid chromatography. Tertiary-butylhydroperoxide (*t*-BHP) was added to human liver-derived HepG2 cells, and the production of reactive oxygen species was measured in the presence and absence of kudzu vine extract. Antioxidant activity was evaluated in all kudzu vine extracts using a hydroxyradical scavenging assay. Thirty-five male Sprague–Dawley rats were divided into seven groups (*n* = 5); two groups were not given any extract or drug, one group was treated with 50 mg/kg silymarin orally for 5 days, and the remaining four groups were respectively treated with 100 mg/kg of 0 %, 30 %, 70 %, or 95 % ethanolic extract of kudzu vine orally once daily for 5 days. On day 5 the treatment groups and one untreated group were fed 0.75 ml/kg carbon tetrachloride (CCl_4_) to induce liver damage. Blood and liver tissue samples were collected 24 h after CCl_4_ administration for measurement of plasma alanine aminotransferase and aspartate aminotransferase, and concentration of malondialdehyde and glutathione in liver tissue.

**Results:**

Puerarin was the most abundant isoflavone in kudzu vine extract. Kudzu vine extract significantly reduced the cytotoxicity and production of reactive oxygen species induced by *t*-BHP in a dose-dependent manner. Treatment with 0 % and 30 % ethanolic extracts of kudzu vine significantly lowered the plasma levels of alanine aminotransferase and aspartate aminotransferase in a CCl_4_-induced hepatotoxicity rat model (*P* < 0.05). Glutathione was significantly elevated in the 30 % ethanolic extract-treated group (*P* < 0.05), while the malondialdehyde level in liver tissue was significantly decreased in the 0 % and 30 % ethanolic extract-treated groups (*P* < 0.05).

**Conclusions:**

The kudzu vine is potentially highly beneficial in treating liver damage, as it scavenges reactive free radicals and boosts the endogenous antioxidant system.

## Background

Kudzu (*Pueraria thunbergiana*) is native to Korea, Japan, China, and India [1], and is now grown worldwide. Kudzu root is used as a source of starch in China and Japan, and is also eaten as a vegetable [2]; it has been used in Traditional Chinese Medicine to treat conditions including the common cold, headache, diarrhoea and heart disease, and is also purported to have various other beneficial effects. Puerarin (4′,7-dihydroxy-8-β-D-glucosylisoflavone) is the major isoflavone compound isolated from kudzu [3, 4]; it has a variety of biological actions in cardiovascular disease, gynaecological disease, osteoporosis, cognitive capability and diabetic nephropathy, including antioxidant activity [3–7].

In contrast to the kudzu root, the kudzu vine is considered to be a waste product with limited usage. The kudzu vine grows one foot per day, outcompeting all other vegetation and making it one of the most noxious invasive plant species in the USA [8, 9]. Kudzu has had the greatest deleterious impact on the forestry industry, where annual productivity losses have been estimated at 100–500 million USD [9].

Recently, kudzu vine has begun to be considered as a new resource. There are currently only few papers investigating the effects of the kudzu vine, including one which reported that kudzu vine effectively reduced bone loss in ovariectomized mice [7]. The kudzu vine contains isoflavones in a similar ratio to those of the kudzu root; hence, the kudzu vine could be a valuable new source of polyphenolic compounds, including isoflavones, which can play an important role as dietary antioxidants [10]. Antioxidants terminate chain reactions by removing free radical intermediates, and inhibit other oxidation reactions by being oxidized themselves. Oxidative intermediates exert their toxic effect through destruction of cellular defence mechanisms [10, 11]. Oxidative stress is a key phenomenon in chronic diseases and hepatotoxicity induced by various chemicals [11]. Until now the hepatoprotective effect of kudzu vine has not been investigated.

The present study was designed to evaluate the effects of kudzu vine extract on hepatic injuries caused by the oxidative stressors tertiary-butylhydroperoxide (*t*-BHP) and carbon tetrachloride (CCl_4_). *t*-BHP has often been used in models to screen the hepatoprotective activity of drugs to investigate the mechanism of cell injury initiated by acute oxidative stress [12], while CCl_4_ is used as a model for studying free radical-induced liver injury and screening hepatoprotective drugs [12, 13].

## Methods

### Preparation of kudzu vine extract

The kudzu vine was collected from Jinan Province, Jeonbuk, Korea in November 2010. The botanical identification was authenticated by Dr Hyoung-Kwon Cho of the Han Poong Pharmaceutical Co., Ltd, where the voucher specimen (#FPR-36) was deposited.

Dried and pulverized kudzu vines (2 kg) were boiled with 2 L of distilled water and various concentrations of ethanol for 3 h. The extracts were concentrated at reduced pressure in a rotary evaporator (N-1000S, EYELA, Japan), yielding a water extract (439.5 g), 30 % ethanolic extract (409.2 g), 70 % ethanolic extract (436.7 g), and a 95 % ethanolic extract (284.8 g).

### High-performance liquid chromatography (HPLC) analysis

The isoflavone contents of the kudzu vine extracts were quantified by HPLC (Waters 2695 system, Waters Co., Milford, MA, USA) using a CAPCELLPAK UG 120 250 × 4.6 mm, 5 μm (Shiseido, Tokyo, Japan). Separation and quantification were achieved at 30 °C using a gradient from solution A (a 10 % aqueous methanol solution with 2 % acetic acid) to solution B (a 98 % aqueous methanol solution with 2 % acetic acid) at a flow rate of 1 ml/min. Peaks were detected at 260 nm. The presence of isoflavones was confirmed by observing a retention time that was the same as that of appropriate standards (Wako Chemical Co., Osaka, Japan).

### *t*-BHP-induced hepatotoxicity in vitro model

#### Cell culture and viability assay

Human liver-derived HepG2 cells were obtained from American Type Culture Collection (Manassas, VA). The monolayer HepG2 cell culture was trypsinized and the cell count was adjusted to 1.0 × 10^5^ cells/ml using Dulbecco’s Modified Eagle Medium containing 10 % foetal bovine serum. 0.1 ml of the diluted cell suspension was added to each well of the 96-well microplate. After 24 h a partial monolayer had formed; the supernatant was removed and the monolayer was washed once with medium. Cultures were treated with *t*-BHP in the presence or absence of kudzu vine extract and incubated for 12 h. Curcumin (20 μM) was used as a reference control. For determination of cell viability, 50 mg/ml 3-[4,5-dimethylthiazol-2-yl]-2,5-diphenyltetrazolium bromide was added to 1 ml of cell suspension (1 × 10^5^ cells per/ml in 96-well plates) for 4 h, and the formazan formed was dissolved in acidic 2-propanol. Optical density was measured at 590 nm.

#### Measurement of reactive oxygen species (ROS)

HepG2 cells (2.5 × 10^4^ cells/ml per 1ml in 24-well plates) were treated with 100 μM of *t*-BHP in the presence or absence of the kudzu vine extract and incubated for 12 h. After washing with phosphate-buffered saline (PBS), the cells were stained with 10 μM 20,70-dichlorofluorescein diacetate in Hank’s balanced salt solution for 30 min in the dark. The cells were then washed twice with PBS and extracted with 1 % Triton X-100 in PBS at 37 °C for 10 min. Fluorescence was measured at an excitation wavelength of 490 nm and an emission wavelength of 525 nm (Spectramax Gemini XS; Molecular Devices, Sunnyvale, CA, USA). Curcumin (20 μM) was used as a reference control.

#### Hydroxyl radical scavenging assay

Hydroxyl radical scavenging assay was performed using the method described by Klouwen [14]. The reaction solution was incubated at 37 °C for 30 min. Absorbance was measured at 520 nm using a UV–Vis spectrophotometer (Spectramax Gemini XS; Molecular Devices, Sunnyvale, CA, USA). The inhibition rate was calculated as follows: [(A_1_- A_2_)/(A_0_- A_2_)] × 100 %, where A_0_ is the absorbance of the control, A_1_ is the absorbance of the sample, and A_2_ is the absorbance of a blank sample. Vitamin C (50 μM) was used as a reference control.

### CCl_4_-induced hepatotoxicity in vivo model

#### Experimental animals and design

Thirty-five male Sprague–Dawley rats (weighing 200–220 g) supplied by Hanil Laboratory Animal Research (Pyeongtaek, Korea) were fed a standard diet (Orient Bio, Korea) and provided with tap water *ad libitum*. This study was approved by the Wonkwang University Animal Care Committee.

**Fig. 1:**
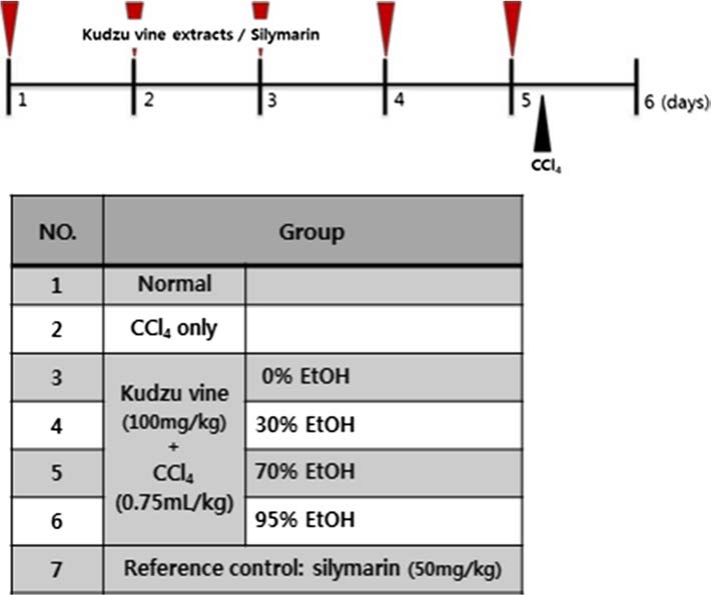
CCl_4_-induced hepatotoxicity in vivo study design. The rats were divided into the following seven groups, with five animals in each group

The rats were divided equally into seven groups (*n* = 5 in each group); two groups were not treated with any extract or drug, while the respective treatment groups were fed either 0 %, 30 %, 70 %, or 95 % kudzu vine extract (100 mg/kg) or the reference drug silymarin (50 mg/kg) for 5 consecutive days. Acute liver injury was induced by a single administration of oral 0.75 ml/kg CCl_4_ diluted in corn oil 1 h to rats in all treatment groups after the final dose of kudzu vine extract or silymarin was administered, and to one of the untreated groups (Fig. 1). The rats were euthanized 24 h later by exsanguination from the abdominal aorta under ether-induced anaesthesia, then blood and liver tissue samples were collected.

#### Estimation of biochemical parameters

The separated plasma was used for the determination of plasma levels of aspartate aminotransferase (AST) and alanine aminotransferase (ALT); the enzyme level was measured according to the method described by Reitman and Frankel [15]. To determine the level of malondialdehyde (MDA), the end product of lipid peroxidation was measured using a thiobarbituric acid reactive substance assay with some modification [16]; the liver was pulverized using a polytron, and then the homogenate was estimated and the supernatant was separated. The total glutathione (GSH) was measured according to the method described by Griffith et al. [17].

### Statistical analysis

Data were expressed as mean ± SD. Significant differences were compared using repeated measures ANOVA followed by the Newman-Keuls multiple range test. Statistical significance was defined as *P* < 0.05. All statistical analyses were performed using SPSS v. 20.0 (IBM, Armonk, NY, USA).

## Results

### Isoflavone composition of kudzu vine extract

**Fig. 2:**
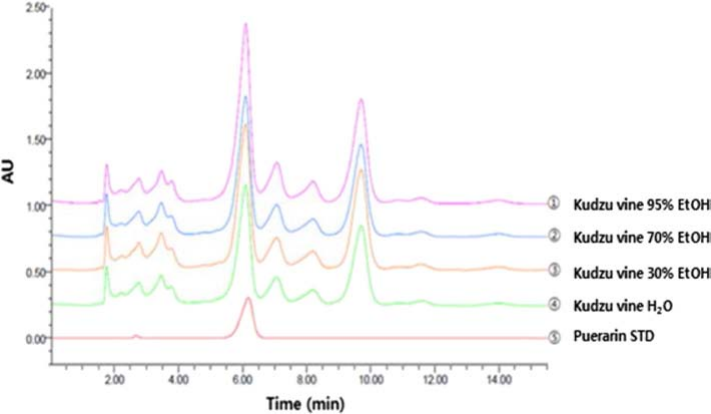
High performance liquid chromatogram of puerarin in crude extract from kudzu vine at 260 nm

Isoflavone contents of kudzu vine extract were quantitated; the isoflavone aglycone and glycoside concentrations in each extract are listed in Table 1. Puerarin was the most abundant isoflavone in kudzu vine extract (Fig. 2).

**Table 1:** Composition of isoflavones in kudzu vine extract

	Puerarin (mg/g)	Daidzin (mg/g)	Glycitin (mg/g)	Genistin (mg/g)	Daidzein (mg/g)	Glycitein (mg/g)	Genistein (mg/g)
H_2_O	236.5	3.33	3.45	3.33	6.27	0.27	0.29
30 % EtOH	303.3	4.33	3.83	4.33	9.22	0.39	0.60
70 % EtOH	288.1	4.26	3.90	4.26	10.1	0.33	0.71
95 % EtOH	372.8	4.50	4.52	4.50	14.9	0.56	1.00

*EtOH* ethanol

### Effects of kudzu vine extract on *t*-BHP-induced hepatotoxicity and free radical scavenging activity

**Fig. 3:**
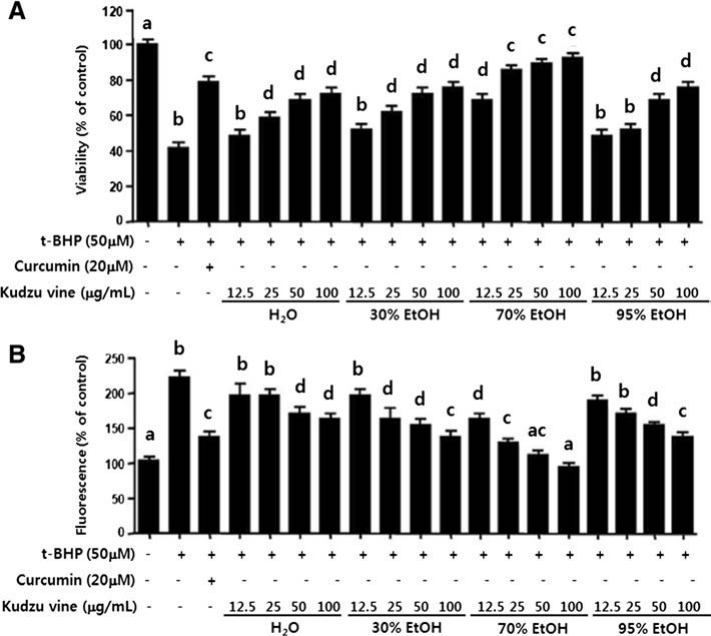
Effects of kudzu vine extract on *t*-BHP-induced (**a**) hepatotoxicity and (**b**) inhibition of ROS generation. The effects of kudzu vine extract on hepatotoxicity and inhibition of ROS generation in *t*-BHP-treated human liver-derived HepG2 cells were investigated. Curcumin was used as a reference control. The results are presented as mean ± SD of three experiments with triplicate samples. Values with different letters (*a*, *b*, *c*, *d*) are significantly different one from another (one-way ANOVA followed by Newman-Keuls multiple range test, *P* < 0.05).

Treatment with *t*-BHP for 12 h increased HepG2 cell death by 2.2-fold compared with untreated cells, while the addition of kudzu vine extracts at non-cytotoxic concentrations (12.5, 25, 50, and 100 μg/ml) caused a significant dose-dependent elevation in cell viability (Fig. 3a).

ROS generation was increased 2.3-fold by *t*-BHP stimulation of hepatocytes. The addition of kudzu vine extracts caused a significant dose-dependent reduction in ROS generation (Fig. 3b). Curcumin was used as a reference control, and treatment with curcumin exhibited significant cytoprotective effects and ROS-scavenging activity [18].

**Fig. 4:**
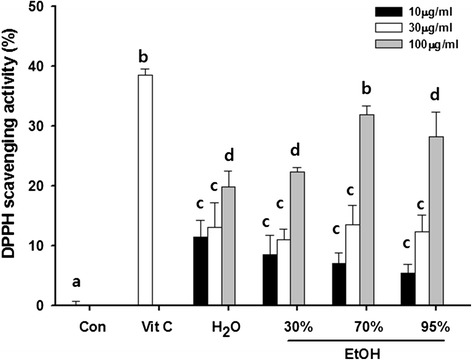
Effect of kudzu vine extract on radical scavenging activity. Vitamin C was used as a reference control. The results are presented as mean ± SD. Values with different letters (*a*, *b*, *c*, *d*) are significantly different one from another (one-way ANOVA followed by Newman-Keuls multiple range test, *P* < 0.05)

The antioxidant activity of the kudzu vine extracts was compared with a representative antioxidant, vitamin C, by hydroxyradical scavenging assay. The kudzu vine extracts at concentrations of 10, 30, and 100 μg/ml exhibited concentration-dependent radical scavenging reactions, from 5.41 to 31.8 % inhibition. The antioxidant activity of 100 μg/ml kudzu vine extract was similar to that of vitamin C (Fig. 4).

### Effect of the kudzu vine extract on markers of hepatic damage

The hepatoprotective effects of the kudzu vine extract are summarized in Table 2. Rats treated with a single dose of CCl_4_ developed hepatic damage. Compared with the normal group, the CCl_4_-treated rats showed a 3-fold increase in plasma ALT (140.0 ± 4.4 unit/ml) and AST (167.3 ± 10.0 unit/ml). The 0 % and 30 % ethanolic extracts significantly blocked the CCl_4_-induced elevation of plasma ALT (106 ± 8.9 and 97.3 ± 4.5 units/ml respectively) and AST (128.8 ± 5.0 and 99.5 ± 4.1 units/ml respectively); similar effects were observed in the silymarin-treated group.

**Table 2:** The effects of kudzu vine extracts on plasma alanine aminotransferase and aspartate aminotransferase levels in CCl_4_-treated rats

Group		Dose (mg/kg)	ALT (units/ml)	AST (units/ml)
Normal			35.4 ± 5.2^a^	60.9 ± 3.2^a^
CCl_4_ only			140.0 ± 4.4^b^	167.3 ± 10.0^b^
Kudzu vine	H_2_O	100	106.1 ± 8.9^c^	128.8 ± 5.0^c^
+ CCl_4_	30 % EtOH	100	97.3 ± 4.5^c^	99.5 ± 4.1^c^
	70 % EtOH	100	152.5 ± 13.7^b^	163.5 ± 9.3^b^
	95 % EtOH	100	151.5 ± 4.9^b^	182.0 ± 8.9^b^
Silymarin		50	100.4 ± 3.8^c^	95.5 ± 4.9^c^

Results presented as mean ± SD. Values with different letters (^a, b, c^) are significantly different one from another (one-way ANOVA followed by Newman-Keuls multiple range test, *P* < 0.05)

### Effect of the kudzu vine extract on hepatic antioxidant markers

**Fig. 5:**
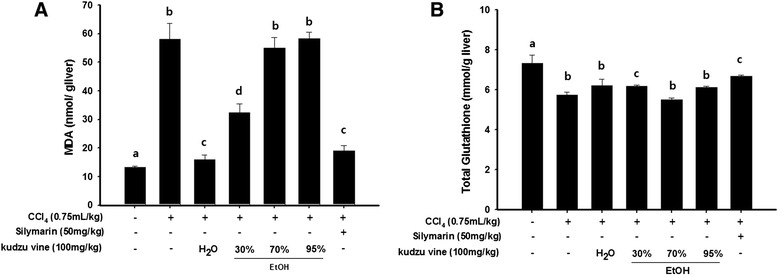
Effects of kudzu vine extracts on hepatic (**a**) malondialdehyde and (**b**) total glutathione amount. The results are presented as mean ± SD. Values with different letters (*a*, *b*, *c*) are significantly different one from another (one-way ANOVA followed by Newman-Keuls multiple range test, *P* < 0.05)

The amount of MDA increased about 4-fold in the CCl_4_-treated group (58 ± 5.6 nmol/g liver) compared with the normal group. However, the 0 % and 30 % ethanolic extracts both significantly inhibited cell damage (MDA 16.0 ± 1.57 and 32.3 ± 3.0 nmol/g liver respectively; Fig. 5a).

The CCl_4_-treated group had significantly reduced GSH levels (5.74 ± 0.1 nmol/g liver) compared with the normal group (7.31 ± 0.4 nmol/g liver). The 30 % ethanolic kudzu vine extract elicited a significant increase in GSH levels (6.17 ± 0.06 nmol/g liver) compared with the GSH levels of the CCl_4_-treated group (Fig. 5b).

## Discussion

The present study found that puerarin was the most abundant isoflavone in kudzu vine extract. In vitro experiments on human liver-derived HepG2 cells showed that kudzu vine extract had cytoprotective and antioxidant properties. In vivo experiments on rats showed that 0 % and 30 % kudzu vine ethanolic extracts significantly reduced hepatic damage (as measured by increase in ALT and AST) and hepatic lipid peroxidation (as measured by increase in MDA), and 30 % kudzu vine extract caused a significant increase in the antioxidant GSH.

Liver disease remains one of the most serious health problems worldwide. The liver is the main organ that metabolizes xenobiotics to help eliminate waste from the body; hence, the liver is exposed to a high concentration of chemicals, drugs, and natural products, which can lead to liver dysfunction, cell injury, and organ failure [19]. More than 100 human diseases, including liver diseases, are related to oxidative stress [20].

Herbal plants have recently gained attention as potential treatments for cancers, metabolic diseases, allergies, ischemia, and inflammation, and especially for their hepatoprotective activities. Most commonly, these hepatoprotective activities seem to be related to the antioxidant capacity of these plants. Naturally derived antioxidants counteract the oxidative stress induced by many hepatotoxins [21]. The quest to discover such naturally occurring antioxidants has become a major scientific focus over the past few decades. The antioxidant activities of isoflavones have been associated with the hepatoprotective effect of these compounds. Plant-derived isoflavone compounds are thought to contribute to the prevention of diseases associated with oxidative stress [22].

Kudzu root is a rich source of isoflavone glucosides. Common isoflavones of kudzu root include puerarin (daidzein 8-C-glucoside), daidzin (daidzein 7-O-glucoside), daidzein, genistein and formononetin [7]. These isoflavones have been associated with antioxidant, hepatoprotective and other pharmacological effects [23, 24]. Among the isoflavones in kudzu root, puerarin is the most abundant (approximately 23% w/w) and has attracted considerable attention because of its potent ability to cause various pharmacological effects [23]. A previous study found that puerarin was the most abundant isoflavone contained in the kudzu vine (nearly 50%), followed by daidzin (3.58%), daidzein (0.92%), genistein (0.03%) and other isoflavones (0.5%) [7]; these results are consistent with the present study. Unlike kudzu root, the beneficial actions of kudzu vine against liver disease have not been extensively investigated.

In the present study, we investigated the antioxidant activity and the hepatoprotective effect of kudzu vine extract. *t*-BHP was used to induce hepatotoxicity in the HepG2 cell line. The cell viability and inhibition of ROS generation was then examined in vitro. All concentrations of kudzu vine extract significantly protected the cell from dying and inhibited the ROS production in a dose-dependent manner. There was a clear correlation between the ratio of protection and the level of antioxidant activity provided by kudzu treatment. ROS production from cells occurs via multiple mechanisms. Large numbers of oxygen free radicals are produced within the liver during the removal of xenobiotics and toxins, and oxidative stress caused by ROS has been linked to various liver diseases [12, 20, 25]. In comparison with normal cells, malignant cells seem to remain functional under higher levels of endogenous oxidative stress in vitro and in vivo [12, 26, 27].

We also used CCl_4_ to induce hepatotoxicity in rats. CCl_4_ is actively converted by cytochrome P-450, especially CYP2E1, in the liver tissues to its highly reactive trichloromethyl free radical CCl_3_^.^. The CCl_3_^.^ radical reacts with cellular macromolecular protein and polyunsaturated fatty acids [12, 13] in the presence of molecular oxygen, it forms more toxic trichloromethylperoxyl radicals CCl_3_O along with H_2_O_2_, O_2_^−^, and OH [28, 29]. So, CCl_4_ is conventionally used to induce liver toxicity, to allow the testing of drugs for their hepatoprotective property. The CCl_4_ treatment elevates the levels of plasma enzymes ALT and AST, which are indicators of cellular leakage and loss of functional integrity of cell membranes in the liver [30, 31]. The activities of ALT and AST were significantly suppressed after the administration of kudzu vine extracts in the present study. This suggests that kudzu vine extract effectively protected hepatocytes against the toxic effects of CCl_4_, resulting in a reduction in plasma ALT and AST levels in CCl_4_-treated rats.

An increase in MDA levels, as seen in our study after CCl_4_ administration, indicates enhancement of lipid peroxidation, leading to tissue damage and failure of antioxidant defence mechanisms to prevent the formation of excessive free radicals [16, 28, 30]. Significant reductions in hepatic lipid peroxidation were detected only in the water and 30 % kudzu vine ethanolic extract-treated groups. There were no significant changes observed in the 70 % and 95 % kudzu vine ethanolic extract-treated groups. Changes in MDA levels and plasma AST and ALT levels displayed the same trends.

GSH was reduced in the liver of CCl_4_-treated rats, while an increased GSH level was observed in conjunction with the administration of 30 % ethanol-derived kudzu vine extract. GSH is a highly effective extra- and intra-cellular antioxidant compound that plays a central role in coordinating the body’s antioxidant defence processes [26]. GSH protects cells against electrophilic attack from xenobiotics such as free radicals and peroxides [28, 29, 32].

Our results suggest that the potent radical scavenging activity of kudzu vine extracts ameliorates oxidative stress and terminates the chain reaction involved in lipid peroxidation. Therefore, the hepatoprotective effect of kudzu vine extract is mainly due to its ability to neutralize the increase of free radicals caused by chemicals. The potency of hepatoprotective activity in high concentration ethanolic extracts was lower than that of the low concentration ethanolic extracts in the rat model studied, indicating that the amount and kind of ingredients may vary depending on the extraction conditions. Further study into the underlying cause of these differences is needed.

## Conclusions

Kudzu vine extracts mitigated the induction of oxidative stress, successfully restored liver function, and significantly alleviated chemical-induced hepatic damage. The kudzu vine might be useful in the prevention of oxidative damage in liver cells and tissues, and should be developed as a new natural drug for the treatment of liver injury by scavenging free radicals and boosting the endogenous antioxidant system.

### Ethics approval and consent to participate

This study was approved by the Wonkwang University Animal Care Committee.

## Abbreviations

ALT: alanine aminotransferase
AST: aspartate aminotransferase
CCl_4_: carbon tetrachloride
GSH: glutathione
HPLC: high-performance liquid chromatography
MDA: malondialdehyde
PBS: phosphate-buffered saline
ROS: reactive oxygen species
*t*-BHP: tertiary-butylhydroperoxide
